# Incidence and Molecular Identification of Begomoviruses Infecting Tomato and Pepper in Myanmar

**DOI:** 10.3390/plants11081031

**Published:** 2022-04-10

**Authors:** Hae-Ryun Kwak, Su-Bin Hong, Hee-Seong Byun, Bueyong Park, Hong-Soo Choi, Si Si Myint, Mu Mu Kyaw

**Affiliations:** 1 Crop Protection Division, National Institute of Agricultural Sciences, Rural Development Administration, Jeonju 55365, Korea; hsubin27@gmail.com (S.-B.H.); hsbyun73@korea.kr (H.-S.B.); florigen1@korea.kr (B.P.); hschoi@korea.kr (H.-S.C.); 2 Department of Agricultural Research (DAR), Ministry of Agriculture, Livestock and Irrigation(MOALI), Yezin, Naypyitaw 15013, Myanmar; sisimyint06@gmail.com (S.S.M.); mumu.kw@gmail.com (M.M.K.)

**Keywords:** begomovirus, leaf curl, yellow mosaic, whitefly

## Abstract

In Myanmar, yellow mosaic and leaf curl diseases caused by whitefly-transmitted begomoviruses are serious problems for vegetables such as tomatoes and peppers. To investigate the incidence of begomoviruses in Myanmar between 2017 and 2019, a field survey of tomato and pepper plants with virus-like symptoms was conducted in the Naypyitaw, Tatkon, and Mohnyin areas of Myanmar. Among the 59 samples subjected to begomovirus detection using polymerase chain reaction, 59.3% were infected with begomoviruses. Complete genome sequences using rolling circle amplification identified five begomovirus species: tomato yellow leaf curl Thailand virus (TYLCTHV), tomato yellow leaf curl Kanchanaburi virus (TYLCKaV), tobacco leaf curl Yunnan virus (TbLCYnV), chili leaf curl Pakistan virus (ChiLCV/PK), and tobacco curly shoot Myanmar virus (TbCSV-[Myanmar]). Excluding the previously reported TYLCTHV, three begomoviruses (ChiLCV/PK, TYLCKaV, and TbLCYnV) were identified in Myanmar for the first time. Based on the 91% demarcation threshold of begomovirus species, TbCSV-[Myanmar] was identified as a new species in this study. Among these, ChiLCV/PK and TbCSV-[Myanmar] were the most predominant in tomato and pepper fields in Myanmar. Identification of begomovirus species may be helpful for predicting the origin of viruses and preventing their spread.

## 1. Introduction

Whitefly-transmitted geminiviruses have emerged as serious constraints to vegetable production, particularly in tropical and subtropical regions [1]. In southeast Asian countries, yellow mosaic and leaf curl diseases caused by begomoviruses (the genus *Begomovirus* in the family *Geminiviridae*) are serious problems in the cultivation of various vegetable crops [2,3]. Begomoviruses are transmitted in a persistent manner by the whitefly *Bemisia tabaci* Gen. (Hemiptera: Aleyrodidae) [4]. The genome of begomoviruses consists of circular single-stranded DNAs, a monopartite or bipartite (designated as DNA-A and DNA-B) genome, which are twinned icosahedral (germinate) particles. Most begomoviruses have bipartite genomes consisting of two DNA components. DNA-A encodes a replication-associated protein, the coat protein (CP), and proteins such as replication enhancer protein and transcription activator protein which participate in the control of replication and gene expression. DNA-B encodes viral-movement proteins. Open reading frames are organized bi-directionally in both genome components and are separated by a non-coding intergenic region containing key elements for replication and transcription [5,6,7]. Several begomoviruses, including most of the tomato yellow leaf curl disease (TYLCV)-associated viruses, have a monopartite genome that resembles DNA-A [8].

According to the begomovirus species demarcation criteria of the International Committee on Taxonomy of Viruses (ICTV), members of different species have a <91% shared nucleotide identity for the full-length genome of monopartite or the full-length DNA-A for bipartite begomoviruses based on pairwise alignments with pairwise deletion of gaps [9]. Currently, 445 begomoviruses are registered in the 2020 report of the ICTV. The representative symptoms of begomoviruses are leaf curling, leaf mosaic, vein yellowing, leaf yellowing, and stunting of plant growth.

In southeast Asia, a wide variety of distinct local begomovirus species has been identified from solanaceous, cucurbitaceous, and malvaceous vegetables [3]. According to the report by Kenyon et al. [2], at least 36 distinct begomovirus species, including TYLCV, have been identified in tomatoes and peppers in southeast and east Asia. Continental southeast Asia appears to be a major center of diversity for begomoviruses, and some species may have spread across the region [2]. Recently, as the damage of yellow mosaic disease attributable to begomoviruses in Asia has been found to be increasing, reports on occurrences of such viruses in several Asian countries such as Bangladesh, Thailand, Indonesia, and India are beginning to emerge [10,11,12,13,14,15,16].

In Myanmar, yellow mosaic and leaf curl diseases caused by begomoviruses pose serious problems for vegetables such as tomatoes and peppers. Nevertheless, except for the tomato yellow leaf curl Thailand virus (TYLCTHV) [2,17], the causal agents are not well-known. Therefore, this study was conducted to investigate the incidence and diversity of begomoviruses infecting tomato as well as pepper crops in Myanmar. A total of 59 samples were collected from tomato and pepper fields in three areas of Myanmar and subjected to PCR using begomovirus detection primers. The result revealed that 35 samples (59.3%) were infected with begomoviruses. Phylogenetic analysis and pairwise sequence identity comparison based on the complete nucleotide sequences of DNA-A for eight begomovirus positive samples identified four additional begomovirus species. Findings from this study will provide useful information for further epidemiological study and the development of effective disease management in tomato and pepper crops of Myanmar.

## 2. Results 

### 2.1. Detection of Begomoviruses in Tomato and Pepper Plants

**Figure 1:**
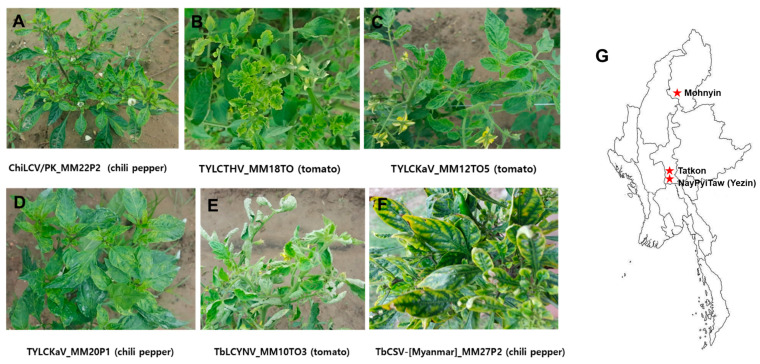
Symptoms observed on tomato and pepper plants collected in Myanmar and a representative map indicating the sampling sites: (**A**) pepper plant showing yellowing, leaf curling, and stunt by ChiLCV/PK (MM22P2), (**B**) tomato plant showing yellowing and leaf curling by TYLCTHV (MM18TO), (**C**) tomato plant showing yellowing and leaf curling by TYLCKaV (MM12to5), (**D**) pepper plant showing yellowing and mosaic by TYLCKaV (MM20P1), (**E**) tomato plant showing yellowing and leaf curling by TbLCYnV (MM10TO3), (**F**) pepper plant showing chlorosis and vein banding by TbCSV-[Myanmar](MM27P2), and (**G**) geographical sites of sampling in Myanmar.

The representative symptoms observed on the infected tomato and pepper samples were yellowing, leaf curling, chlorosis, vein banding, mosaic, yellow mosaic, deformation of leaves, and stunting of plants (Figure 1A–F). A total of 59 tomato and pepper leaf samples collected in the Naypyitaw, Tatkon, and Mohnyin areas of Myanmar (Figure 1G) were tested for begomoviruses by PCR using detection primers that amplify the encoding coat protein. Of these, 35 samples (59.3%) were found to be infected with begomoviruses which were contributed by 19 of 36 tomato samples (52.8%) and 16 of 23 chili pepper samples (69.6%) (Table 1). In accordance with earlier reports, the conserved region of the begomovirus AV1 gene encoding CP was used for provisional identification [18,19]. The amplified PCR products were sequenced and analyzed by BLASTn (NCBI). BLASTn analysis of the CP gene partial sequences of DNA-A revealed that 33 begomovirus-positive samples had nucleotide sequence identities (93–99%) with the four previously known begomovirus species: chili leaf curl Pakistan virus (ChiLCV/PK), tomato yellow leaf curl Thailand virus (TYLCTHV), tomato yellow leaf curl Kanchanaburi virus (TYLCKaV), and tobacco leaf curl Yunnan virus (TbLCYnV). Two samples had relatively very low sequence identities of 79% with tobacco leaf curl Pusa virus (TbLCPV) (Table 1).

### 2.2. Pairwise Sequence Comparison and Phylogenetic Analysis of Begomovirus Species 

**Figure 2:**
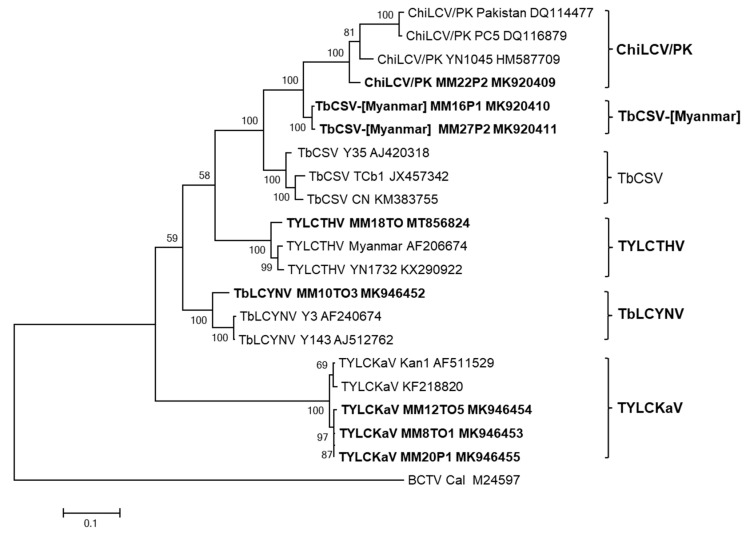
Phylogenetic analysis based on the complete nucleotide sequences of monopartite or DNA-A of eight begomovirus isolates from pepper and tomato plants in Myanmar and the corresponding sequences of begomoviruses previously reported in GenBank. The phylogenetic tree was reconstructed by maximum likelihood in MEGA X using the TN93+G model. The bootstrap values are indicated at the nodes (based on 1000 replicates). Viruses isolated in this study are shown in bold font. The tree was rooted using *Beet curly top virus* (BCTV: accession no. M24597) as an outgroup.

**Figure 3:**
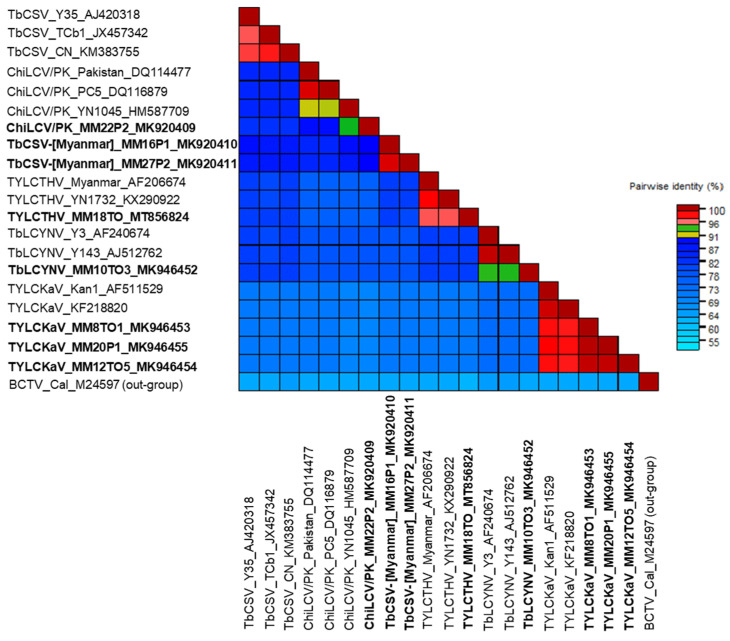
Color-coded pairwise identity matrix generated by Sequence Demarcation Tool from 20 begomovirus genomes. BCTV (accession no. M24597) was used as an outgroup for the analysis. Each colored cell represents a percentage identity score between two sequences (red = 94% {strains}, yellow-green = 91% {same species}, blue < 91% {different species}). Viruses isolated in this study are shown in bold font.

To establish new species and a definite classification, full-genome sequences of DNA-A are needed [9]. In this study, the genomes of eight isolates of four begomoviruses (ChiLCV/PK, TYLCTHV, TYLCKaV, and TbLCYnV) preliminarily identified through CP sequencing from tomato and pepper plants were completely sequenced using RCA and the results were deposited in GenBank (Table 2). The complete genome nucleotide sequences of DNA-A of eight begomovirus isolates were compared to those of previously reported begomovirus isolates. Phylogenetic analysis (Figure 2) and pairwise sequence identity comparison (Figure 3), based on the complete nucleotide sequences of DNA-A, showed that four viruses belonged to the same clade with closely related begomoviruses at the highest nucleotide sequence identities. TYLCTHV isolate MM18TO from tomato plants showed 96% nucleotide sequence identity to previously reported Myanmar isolate (AF206674). Three TYLCKaV isolates, MM8TO1 and MM12TO5 from tomato plants and MM20P1 from pepper plants, clustered in the same group and had maximum nucleotide sequence identities of 98% to isolate Kan1 from Thailand (AF511529). A TbLCYnV isolate, MM10TO3 from tomato plants, had a nucleotide sequence identity of 93% to isolate Y143 from China (AJ512762). The ChiLCV/PK isolate MM22P2 from pepper plants had a nucleotide sequence identity of 94% to isolate YN1045 from China (HM587709). However, isolates MM16P1 and MM27P2 from pepper plants, which were preliminarily identified as ChiLCV/PK, formed an overlapping complex group at less than 90% nucleotide identities with tobacco curly shoot virus (TbCSV) and ChiLCV/PK isolates. Based on the 91% demarcation threshold, these virus isolates can be considered as new candidate begomovirus species, and we propose the provisional name tobacco curly shoot Myanmar virus (TbCSV-[Myanmar]).

### 2.3. Molecular Characterization of TbCSV-[Myanmar] Isolates 

To confirm begomovirus isolates MM16P1 and MM27P2 as new candidate begomovirus species, the complete nucleotide sequence and amino acid sequences of six encoded proteins were compared with the reference sequences from NCBI the database (Table 3). The complete nucleotide sequences between MM16P1 and MM27P2 isolates had 99% sequence identity. Pairwise nucleotide sequence comparison revealed that MM16P1 and MM27P2 shared the highest identity at 85–86% with TbCSV isolates and shared 79–82% identities with ChiLCV/PK isolates. Multiple alignment and comparison of individual proteins with begomoviruses showed that MM16P1had the highest amino acid sequence identities of 96–97% for V1 (CP) and 92–96% for V2 (MP) of TbCSV and ChiLCV/PK isolates. While MM16P1 had amino acid sequence identities of 83–90% for C1 (Rep), C2 (TrAP), C3 (Ren), and C4 proteins of TbCSV isolates, it had amino acid sequence identities of 90–97% for C2 (TrAP) and C3 (Ren), 77–80% for C1 (Rep), and the lowest identities of 32–35% for C4 proteins of ChiLCV/PK isolates. The phylogenetic analyses and amino acid sequence comparison provided the possibility of recombination events in the MM16P1 and MM27P2 DNA-A. To examine the occurrence of recombination events in the MM16P1 and MM27P2 DNA-A, we used seven recombination analysis methods: RDP, GENECONV, BootScan, MaxChi, Chimaera, SiScan, and 3Seq in the RDP v4.101 program, and recombination events detected by at least six different methods were considered reliable. The result showed that at least one significant recombination event had occurred in MM16P1 and MM27P2 DNA-A, between nucleotides 16 and 2106. TbCSV-CN[BD:Raj:02:25:Tom:10] was identified as the major parent, and ChiLCV/PK-YN1045 was identified as the minor parent (Table 4).

### 2.4. Diversity of Begomoviruses in Tomato and Pepper

**Figure 4:**
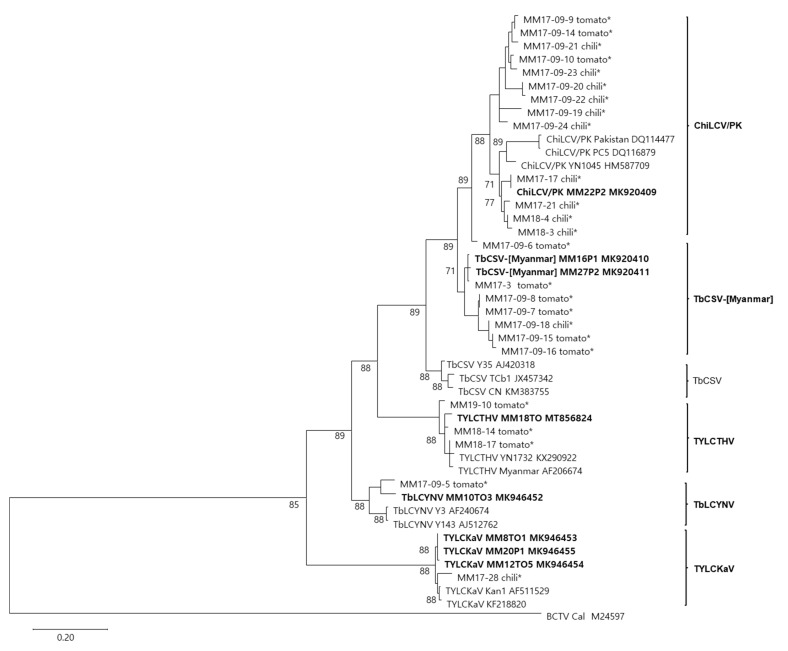
Phylogenetic analysis based on the nucleotide sequences of the CP gene of 33 isolates of begomoviruses collected from tomato and pepper plants in this study and the corresponding sequences of begomoviruses previously reported in GenBank. Phylogenetic trees were reconstructed by maximum likelihood in MEGAX using the TN93 + G model. The bootstrap values are indicated at the nodes (based on 1000 replicates) and only bootstrap values greater than 50% are shown. The tree was rooted using *Beet curly top virus* (BCTV: accession no. M24597) as an outgroup. Viral isolates for which the DNA-A genome was sequenced in this study are shown in bold. The other viral isolates for which the partial CP genes were sequenced in this study are indicated by asterisks as ‘isolate name-host plant’ and their sequences are given in the supplementary file.

The CP coding nucleotide sequences of 35 begomovirus-positive samples, which include 8 isolates identified as 5 begomovirus species, were aligned with the corresponding sequences of their closely related begomoviruses in the GenBank database. The phylogenetic tree showed that 33 begomovirus-positive isolates, except for 2 unidentified isolates from tomato, clustered with four previously reported begomovirus species (ChiLCV/PK, TYLCTHV, TYLCKaV, and TbLCYnV) and a potentially new begomovirus species proposed in this study (TbCSV-[Myanmar]) (Figure 4). Among the 23 isolates preliminarily identified as ChiLCV/PK by BLASTn analysis of CP partial sequences, 8 isolates were clustered with 2 TbCSV-[Myanmar] isolates. Considering these data, the distribution and diversity of begomoviruses infecting tomato and pepper in Myanmar were summarized in Table 1. In pepper plants, three begomoviruses, ChiLCV/PK, TbCSV-[Myanmar], and TYLCKaV, were identified in Myanmar for the first time. Tomato plants in Myanmar were infected with relatively more diverse begomoviruses. Aside from the previously reported tomato-infecting begomovirus (TYLCTHV), four additional begomoviruses (ChiLCV/PK, TbCSV-[Myanmar], TYLCKaV, and TbLCYnV) were identified in tomatoes for the first time. Among these, ChiLCV/PK and TbCSV-[Myanmar] were the most common begomoviruses in tomato and pepper fields in Myanmar. For accurate species classification, the remaining isolates need to be completely sequenced. In addition, the unidentified begomovirus from two tomato samples (78% identity with TbLCPV) needs to be studied.

## 3. Discussion

Tomato (*Solanum lycopersicum*) and pepper (*Capsicum annuum*) plants belong to the family *Solanaceae* and are major vegetable crops in tropical and sub-tropical regions of the world. In Myanmar, during the year 2017/18, tomatoes and peppers (dried) were cultivated on 103,000 and 108,000 hectares with a total production of 1,157,000 and 130,000 metric tons, respectively (Myanmar agriculture at a glance, 2019). In general, tomato and pepper production are severely affected by various viral diseases. Begomovirues are the major group of pathogens that cause yellow mosaic and leaf curl disease. At least 36 distinct species of begomovirus, including TYLCV, have been identified from tomato and pepper plants in southeast and east Asia [2]. Studies on the identification of begomovirus species may be helpful to predict the origin of these viruses and prevent their spread. 

In this study, the diversity of begomoviruses in tomato and pepper crops in Myanmar was examined for the first time. PCR using the universal begomovirus primers was performed for the detection of begomovirus, and the complete genome sequences using RCA identified four previously known begomovirus species and one new candidate begomovirus species. Prior to this study, only one begomovirus, TYLCTHV, had been reported in tomatoes [2,17] and no begomovirus was reported in peppers from Myanmar. In the present study, four additional begomoviruses (ChiLCV/PK, TbCSV-[Myanmar], TYLCKaV, and TbLCYnV) in tomato fields and three begomoviruses (ChiLCV/PK, TbCSV-[Myanmar], and TYLCKaV) in pepper fields were identified in Myanmar for the first time. 

Infection by TYLCTHV, ChiLCV/PK, TYLCKaV, and TbLCYnV have been reported in several countries in east and southeast Asia. TYLCTHV, as a bipartite begomovirus, was first identified in tomatoes from Thailand and has since been reported in Cambodia, China, and Taiwan [2,20]. TYLCKaV, as a bipartite begomovirus, was first identified in tomatoes and eggplants in the Kanchanaburi province of Thailand [21] and has also been reported to infect tomatoes, eggplants, and peppers in Laos, Vietnam, Cambodia, and Indonesia [2,22,23,24,25]. TbLCYnV, as a monopartite begomovirus, was first identified in tobacco in Yunnan province of China [26], and then detected in tomato plants in Thailand [11]. ChiLCV/PK is classified as a strain of chili leaf curl virus and is one of the causative viruses of chili Leaf curl disease (ChiLCD). ChiLCVD is the most destructive virus in terms of incidence and yield loss [27]. ChiLCV, as a monopartite begomovirus, has been reported in several crops including peppers, tomatoes, watermelons, and squash in different countries such as Pakistan, India, and Oman [28,29,30,31,32]. ChiLCV has been spreading into different geographic areas to infect different host plants. 

TbCSV-[Myanmar], the new candidate begomovirus species proposed in this study, exhibited a maximum identity of 86% with a TbCSV isolate (Y35:2001:AJ420318) reported in China. TbCSV, as a monopartite begomovirus, has been reported in tobacco, tomato, pepper, watermelon, and common bean plants [33,34,35,36]. Some TbCSV isolates were identified to associate with a betasatellite (tobacco curly shoot betasatellite, TbCSB), which is not necessary for infection but intensifies symptoms [36,37]. However, no betasatellites were detected in TbCSV isolates from peppers [34]. In the case of the TbCSV-[Myanmar] isolates, to determine whether a satellite was associated with these isolates, a universal betasatellite-universal primer pair (CLB36F/CLB37R) was used [38] which did not show any amplified products, indicating their absence. Moreover, no alphasatellite or possible DNA-B component was detected by digestion of the RCA products. To establish the status of this new candidate begomovirus, further studies on pathogenicity, host range, and whitefly transmission are required. 

Mixed infections of begomoviruses have been reported to occur frequently in tomato and pepper crops worldwide [11,24]. However, in this study, mixed infections by begomoviruses were not observed. It is necessary to determine whether mixed infection through begomovirus-species specific primers is present.

The diversity of begomoviruses obtained in this study will provide useful information for further epidemiological studies and the development of effective disease management strategies for tomato and pepper crops of Myanmar. Additionally, these data can be applied to the development of diagnostic assays to detect begomoviruses specifically for continuous monitoring of viral diversity in various economic crops. 

## 4. Materials and Methods

### 4.1. Sample Collection 

To investigate the incidence of begomoviruses, a total of 59 leaf samples with virus-like symptoms were collected from 36 tomato (*Lycopersicon esculentum*) and 23 pepper (*Capsicum annuum* L.) plants in Naypyitaw (19°82′ N; 96°27′ E), Tatkon (20°13′ N; 96°21′ E), and Mohnyin (24°47′ N 96°22′ E) areas of Myanmar (Figure 1G; Table 1). Samples were collected as dried leaf samples or using Biocube (Biocubesystem, Suwon, Korea) from leaf tissues according to the manufacturer’s instructions. The Biocube, which is a porous ceramic cube with a pore size of about 160–340 nm, can rapidly absorb nucleic acid fragments into its pores and be added directly into a polymerase chain reaction (PCR) tube as a template without any solvent extraction process [39]. When samples could not be transported directly, due to quarantine, or when it was difficult to maintain freshness for a long time, virus-infected samples are collected using a Biocube folder. Dried leaf samples were initially stored at 2–8 °C and later at −70 °C, whereas the Biocube samples were stored with desiccants in zipper-seal bags at room temperature. 

### 4.2. Total Nucleic Acids Extraction

Total DNA was extracted from infected leaf samples using the Viral Gene-spin^TM^ viral DNA/RNA extraction kit (Intron, Seongnam, Korea) according to the manufacturer’s instructions. The Biocube-absorbed sap of leaf tissue samples was used directly as a template for PCR or heat-treated at 95 °C for 5 min in 20 µL of distilled water and then 1 μL was taken as a template. 

### 4.3. PCR, Cloning, and Sequencing

For begomovirus detection, PCR reactions were carried out using the begomovirus detection primer pairs (uni2-F: 5′-TGTGAAGGCCCATGTAAGGTCCAGTC-3′, uniB-R: 5′-ACAGGGTTAGAGGCATGAGTACATGCC-3′), amplifying the partial coat protein gene of 524 nucleotides [40]. PCR reactions were performed in a volume of 20 μL containing 1 μL total nucleic acids (or Biocube or 1 μL of eluate from Biocube), 1 μL of each primer (10 μM), 1 μL dNTPs (10 mM mix), 2 μL MgCl_2_ (25 mM), 4 μL 5 × reaction buffer, 0.5 U/μL Go-Taq DNA polymerase (Promega, Madison, WI, USA), and nuclease-free water. Amplification was carried out under the following cycling conditions: initial denaturation at 95 °C for 2 min; 35 cycles of denaturation at 95 °C for 20 s, annealing at 55 °C for 30 s, and extension at 72 °C for 1 min; followed by an incubation period at 72 °C for 5 min. The PCR products were analyzed by electrophoresis in 1.0% agarose gel at 100 V for 60 min, staining was performed with 5 μL/100 mL EcoView (MorningBio, Jeonju, Korea), and DNA bands were visualized using a UV transilluminator. The PCR fragments were purified using a MEGAquick-spin™ Kit (Intron, Seongnam, Korea) and cloned into the pGEM-T easy vector (Promega, Madison, WI, USA) according to the manufacturer’s instructions, followed by transformation into *Escherichia coli* DH5α. For the complete genome sequences, rolling circle amplification (RCA) was performed using the TempliPhi kit (GE Healthcare Life Sciences, Uppsala, Sweden). The RCA amplified product was digested by restriction enzyme *Bam*HI and separated on 1% agarose gel. DNA fragments (approximately 2.8 kb) purified from the gel were cloned into the *Bam*HI-digested RBC T&A vector (**RBC** Bioscience, Taipei, Taiwan). The clones of each fragment were sequenced completely by a commercial company (Bionics, Daejeon, Korea). The resultant sequences were assembled using SeqMan of the DNAStar v. 5.02 program (Lasergene, Madison, WI, USA) and were submitted to the GenBank database.

### 4.4. Sequence and Phylogenetic Analyses

The nucleotide sequences obtained were analyzed using the BLASTn at the NCBI website to identify species having highly similar sequences according to the recommendation of the Geminiviridae Study Group of the ICTV. Virus sequences were aligned using the ClustalW tool [41] and compared with those of previously reported isolates (Table 2). The phylogenetic relationships of the begomovirus sequences were analyzed by the maximum likelihood method in MEGAX [42]. In the maximum likelihood analyses, the phylogenetic trees were constructed using best fit nucleotide substitution models (TN93 + G the CP region, and the full-length genome). Bootstrap values were calculated using 1000 random replications. All positions containing gaps and missing data were eliminated. Monopartite genome sequence of *Beet curly top virus* (BCTV: accession no. M24597: *Curtovirus* genus) was used as an outgroup. To determine the pairwise nucleotide identity, the Sequence Demarcation Tool v. 1.2 program based on muscle alignment was used [43]. Each colored cell represents a percentage identity score between two sequences (red = 94% {same strains}, yellow-green = 91% {same species}, blue < 91% {different species}).

### 4.5. Recombination Analysis of TbCSV-[Myanmar] Isolates

To examine whether recombination events occurred in the TbCSV-[Myanmar] isolates, we used seven recombination analysis methods: RDP, GENECONV, BootScan, MaxChi, Chimaera, SiScan, and 3Seq in the RDP v4.101 (Recombination Detection Program, ver. 4) program [44]. The parameter was set to default options following the manufacturer’s recommendation. Then recombination events were computed with the highest acceptable *p*-Value set to 0.01.

## Figures and Tables

**Table 1:** Tomato and pepper plants collected from Myanmar and detection of begomovirus.

Surveyed Vegetable Crops	Collected Year	Collected Area	No. Of Collected Samples	No. of Plant Positive for Begomovirus By PCR	No. of Begomovirus Species Identified Based on Blast Analysis of Amplified Product ^a^			
					ChiLCV/PK	TYLCTHV	TYLCKaV	TbLCYnV
Tomato	2017	Naypyitaw	9	7 ^b^	1 (1) ^c^	1	2	1
	2017	Naypyitaw	15	9	8 (5) ^c^			1
	2018	Naypyitaw	3	1		1		
	2018	Tatkon	4	1		1		
	2018	Mohnyin	2	0				
	2019	Naypyitaw	3	1		1		
Subtotal			36	19 (52.8%)	9 (6) ^c^	4	2	2
Pepper	2017	Naypyitaw	5	5	4 (1) ^c^		1	
	2017	Tatkon	2	2	1 (1) ^c^		1	
	2017	Naypyitaw	9	7	7 (1) ^c^			
	2018	Naypyitaw	3	0				
	2018	Tatkon	4	2	2			
Subtotal			23	16 (69.6%)	14 (3) ^c^		2	
Total			59	35 (59.3%)	23 (9) ^c^	4	4	2
Identities of the closely reported begomovirus in Genbank					94–96%, HM587709	96–99%, KX290922	98%, AF511529	93%, AF240674

^a^ Species were identified based on BLAST analysis of amplified products including the CP gene. All isolates had nucleotide sequence identities of greater than 93% of the reference sequences reported in GenBank. ChILCV/PK, *Chlli leaf curl Pakistan virus*; TYLCTHV, *Tomato yellow leaf curl Thailand virus*; TYLCKaV, *Tomato yellow leaf curl Kanchanaburi virus*; TbLCYnV, *Tobacco leaf curl Yunnan virus*. ^b^ Including two unidentified begomoviruses. ^c^ No. of a tentatively new begomovirus species, TbCSV-[Myanmar], by phylogenetic analysis based on CP partial and complete genome sequences of DNA-A.

**Table 2:** Database of the complete nucleotide sequences of monopartite or DNA-A of begomoviruses isolated from Myanmar and reference sequences.

Virus ^a^	Isolate ^b^	Host Plant	Origin	Genome (nt)	NCBI Accession No.
ChiLCV/PK	**MM22P2**	Pepper	Myanmar: Naypyitaw	2759	MK920409
	YN1045	Pepper	China	2752	HM587709
	Pakistan	Pepper	Pakistan	2756	DQ114477
	PC5	Pepper	Pakistan	2756	DQ116879
TYLCTHV	**MM18TO**	Tomato	Myanmar: Naypyitaw	2747	MT856824
	YN1732	Tomato	China	2744	KX290922
	D[MY:Yan:99]	Tomato	Myanmar	2746	AF206674
TYLCKaV	**MM8TO1**	Tomato	Myanmar: Naypyitaw	2753	MK946453
	**MM12TO5**	Tomato	Myanmar: Naypyitaw	2753	MK946454
	**MM20P1**	Pepper	Myanmar: Naypyitaw	2753	MK946455
	TH:Kan1	Tomato	Thailand	2752	AF511529
	Laos	Eggplant	Laos	2752	KF218820
TbLCYnV	**MM10TO3**	Tomato	Myanmar: Naypyitaw	2753	MK946452
	Y3	Tobacco	China	2744	AF240674
	Y143	Tobacco	China	2750	AJ512762
TbCSV	Y35	Tobacco	China	2746	AJ420318
	CN[BD:Raj:02:25:Tom:10]	Tomato	Bangladesh	2746	KM383755
	TCb1	Tomato	India	2758	JX457342
TbCSV-[Myanmar]	**MM16P1**	Pepper	Myanmar: Naypyitaw	2762	MK920410
	**MM27P2**	Pepper	Myanmar: Tetkone	2762	MK920411

^a^ ChILCV/PK, Chili leaf curl Pakistan virus; TYLCTHV, Tomato yellow leaf curl Thailand virus; TYLCKaV, Tomato yellow leaf curl Kanchanaburi virus; TbLCYnV, Tobacco leaf curl Yunnan virus; TbCSV-[Myanmar], Tobacco curly shoot Myanmar virus, a new species proposed in this study. ^b^ Isolates analyzed in this study are shown in boldface.

**Table 3:** Nucleotide and amino acid sequence identities (%) between Myanmar isolates TbCSV-[Myanmar]-MM16P1 and other TbCSV and ChiLCV/PK isolates.

Virus Isolate	NCBI Accession No.	Full-Genome Nucleotide Sequence Identities (%)	Amino Acid Sequence Identities (%)					
			V1 (CP)	V2 (MP)	C1 (Rep)	C2 (TrAP)	C3 (Ren)	C4
TbCSV-[Myanmar]-MM27P2	MK920411	99	99	100	98	97	96	96
TbCSV-Y35	AJ420318	86	97	94	88	84	83	90
TbCSV-TCb1	JX457342	85	96	93	87	86	84	89
TbCSV-CN[BD:Raj:02:25:Tom:10]	KM383755	85	96	92	87	86	83	90
ChiLCV/PK-MM22P2	MK920409	80	98	96	80	97	93	32
ChiLCV/PK-YN1045	HM587709	82	98	94	78	92	90	35
ChiLCV/PK-Pakistan	DQ114477	79	97	93	77	91	91	32
ChiLCV/PK-PC5	DQ116879	80	98	94	77	90	91	33

**Table 4:** Detection of possible recombination in the TbCSV-[Myanmar] DNA-A sequence.

Recombination Breakpoints	Parental Isolates ^a^		RDP4 (*p*-Value) ^b^
	Major	Minor	
16-2106	TbCSV-CN[BD:Raj:02:25:Tom:10]	ChiLCV/PK-YN1045	R, G, B, M, C, S, 3S (4.895 × 10^−39^)
	(KM383755)	(HM587709)	

^a^ ‘Parental isolate’ denotes the most likely isolate among analyzed isolates; Major parent × minor parent. ^b^ RDP4-implemented methods supporting the corresponding recombination sites; R (RDP), G (GENECONV), B (BootScan), M (MaxChi), C (Chimaera), S (SiScan), and 3S (3Seq). The reported *p*-value in parentheses is the smallest *p*-value among the calculated *p*-values using RDP4-implemented methods and the corresponding method is shown in boldface.

## Data Availability

The data is contained within the article or supplementary material. The genome sequence of begomovirses in this study have been deposited in the NCBI database.

## Supplementary Materials

The following supporting information can be downloaded at: https://www.mdpi.com/article/10.3390/plants11081031/s1, The nucleotide sequences for partial CP gene of begomovirus isolates obtained from this study.
